# Chronicity of sleep problems in children with chronic illness: a longitudinal population-based study

**DOI:** 10.1186/1753-2000-3-22

**Published:** 2009-08-27

**Authors:** Børge Sivertsen, Mari Hysing, Irene Elgen, Kjell Morten Stormark, Astri J Lundervold

**Affiliations:** 1 Department of Clinical Psychology, University of Bergen, Bergen, Norway; 2 Department of Biological and Medical Psychology, University of Bergen, Norway; 3 Department of Pediatrics, Haukeland University Hospital, Bergen, Norway; 4 Centre for Child and Adolescent Mental Health, Unifob Health, Bergen, Norway

## Abstract

**Background:**

The aim of this study was to examine the chronicity of sleep problems in children with chronic illness, and potential predictors of sleep problems.

**Methods:**

Using data from a longitudinal total population study in Norway, The Bergen Child Study, data on sleep problems, chronic illness and potential confounders were assessed at ages 7-9 and 11-13.

**Results:**

295 of 4025 (7.3%) children had a chronic illness, and the prevalence of chronic sleep problems was significantly higher in this group compared to children without chronic illness (6.8% versus 3.6%). Sleep problems at the first wave increased the risk of sleep problems at the second wave, also when adjusting for potential confounders (odds-ratio = 5.41). Hyperactivity and emotional problems were also independent risk factors for later sleep problems.

**Conclusion:**

These findings call for increased awareness and development of treatment strategies of sleep problems in children with chronic illness.

## Background

Sleep problems are among the most common complaints in children, and have been linked to a range of negative consequences, including reduced daytime functioning, academic and cognitive deficits as well as increased risk of emotional and behavioural problems [1,2]. Children with chronic illness are at increased risk for sleep problems, and several cross-sectional studies have found an increased rate of sleep problems in children with specific chronic illnesses, including cerebral palsy [3], epilepsy [4], asthma [5], headaches [6], and migraine [7]. In one of the few population-based studies assessing sleep problems among children with chronic illness, Hysing et al. [8] found that these children reported more problems falling asleep and had more night-time awakenings compared to their healthy peers.

Few longitudinal studies of children in the general population have explored the stability of sleep problems, and with mixed findings. In a Swiss study [9] following children from infancy to 10 years, night-time awakenings were found to be both frequent and persistent over time. In contrast, Gregory et al. [10] found a reduction of sleep problems from early childhood to mid-adolescence, and Laberge et al. [11] found a similar reduction in sleep onset problems in children from 10 to 13 years. However, little is known with regards to the chronicity of sleep problems in children with chronic illness, and to the best of our knowledge, no longitudinal population-based studies have investigated the stability of sleep problems over time in this group of children.

The increased rate of sleep problems in children with chronic illness may have several potential pathways, some of them suggesting a higher likelihood of chronicity. For example, chronic illness may affect the sleep physiology and sleep systems in disorders with impaired central nervous system (CNS) functioning. Other factors contributing to a chronic trajectory of sleep problems in chronic illness may include higher rates of upper-airway obstruction and BMI (body mass index), as well as emotional and behavioural disorders, which previously has been linked to sleep problems in children with chronic illness [8]. It is also possible that parental stress related to managing their child's chronic illness might contribute to poor implementation of sleep schedules, and thus sleep problems.

Based on the same study population as the study by Hysing et al. [8], the current paper linked two waves of the Bergen Child Study (BCS), assessing all children at two time points (7-9 and 11-13 years of age) in order to explore the chronicity of sleep problems in children with chronic illness. We hypothesized that children with chronic illness would report higher rates of both acute and chronic sleep problems than their peers, and that sleep problems would differ between specific subgroups of chronic illnesses. We expected both sleep problems and behavioural and emotional problems to predict subsequent sleep problems.

## Methods

### Study design and subjects

Data stem from the first and second wave of the BCS, carried out in the fall 2002 and spring 2006, respectively. The BCS is a longitudinal total population-based study of children in all public and private schools in the city of Bergen, Norway. The protocol and population of the BCS is described in detail elsewhere [8,12]. In short, in the *first wave*, the target population was 9430 primary school children aged 7 to 9 years, of which 7007 parents gave their informed consent to participate, yielding a response rate of 74.3%. The *second wave *was conducted in 2006, and in all 5196 children, now aged 11 to 13 years, participated (response rate: 55.1%). A total of 4025 children participated in both waves. In all, 387 children were reported by their parents to have a chronic illness in the second wave. The 295 (7.3%) children who were identified to have such an illness in both waves were included in the present study.

### Instruments

#### Chronic illness (wave 2 only)

Chronic illness (CI) was defined the following way: All parents responded to a simple question in wave 2 of the BCS regarding whether or not their child had a chronic illness or a disability. Parents who rated such illness/disability as present went on to categorize it as either (1) asthma, (2) epilepsy, (3) diabetes, (4) mental retardation or (5) other illnesses. Parents who endorsed other illness were asked to specify in their own words what that illness was. Of the 5683 children, 387 (9.6%) were reported to have at least one CI. An experienced paediatrician (IE) categorized the illness in subgroups. In the present study three subgroups of chronic illness were identified and included; somatic illness, neurological illness and asthma. Due to the overlap between children with asthma and allergy/eczema, the children where the parents only reported allergy/eczema were excluded. Thus, CI was defined as reported by parents and only somatic disorders were included (see Table 1 for all included illnesses). Children reported to have psychiatric disorders (*n *= 25) and specific learning disabilities (*n *= 6) on the question about physical illness were included in the non-chronically ill group for statistical analyses. Children with more than one chronic illness were categorized to one illness group in the following order: neurological disorders, asthma and somatic illness. Note that children may have more than one diagnosis.

**Table 1:** Sub-groups of chronic illness in the second wave of the Bergen Child Study*

Subgroups (n)		n
Neurological disorders (76)	Mental retardation and related syndromes	27
	Epilepsy	20
	Migraine	13
	Cerebral palsy	6
	Hydrocephalus and myelomeningocele	4
	Other	6
Asthma (188)		
Somatic disorders (55)	Diabetes	14
	Gastrointestinal disorders	14
	Skeletal disorders	12
	Cardiovascular disorders	3
	Heamophiliac	3
	Kidney	3
	Endocrinological disorders	3
	Muscle disorders	2
	Rheumatism	1

* Children may have more than one chronic illness

#### Emotional and behavioural disorder (wave 1 and 2)

The Strengths and Difficulties Questionnaire (SDQ) [13,14] is a behavioural screening questionnaire for children aged 4-16 years comprising 25 items, which can be allocated to five subscales with five items each: (1) emotional symptoms, (2) conduct problems, (3) hyperactivity-inattention problems, (4) peer relationship problems and (5) pro-social behaviour. A total difficulty score is computed by combining the first four subscale scores. Each subscale is scored on a three-point scale; 'not true', 'somewhat true', and 'certainly true', with total subscale scores each ranging from 0-10, and total difficulties score from 0-40. The SDQ has been extensively validated in various countries (e.g. in population studies of children and adolescents in Nordic countries) [15-17]. The SDQ was completed by the parents in wave 1, whereas in wave 2 the SDQ was provided also by the children.

#### Sleep problems (wave 1 and 2)

Child-reported sleep problems were assessed with one question encompassing difficulties with initiating and/or maintaining sleep (DIMS: "Does your child have problems initiating sleep or have frequent awakenings"), rated on a three-point Likert scale ("completely correct" "partly correct" and "not correct"). A dichotomous variable was used for the purposes of the present study, in which responding either "completely correct" or "partly correct" was defined as having DIMS. No data on the time-frame or severity of the sleep problems were available. This operationalization has previously been applied in the BCS [18]. Chronic sleep problems were defined as reporting DIMS at both waves, whereas transient (acute) sleep problems were defined as reporting DIMS at either of the two waves.

#### Demographical/clinical information (wave 2 only)

Level of the parental education was reported in three categories (primary school, secondary school and college/university), while household economy was rated as good, medium or poor by the parents. The child's body mass index (BMI) was calculated as weight (kg) divided by squared height (cm). For the purposes of the present study we used the following percentiles: "underweight": Less than the 5th percentile, "healthy weight": 5th percentile to less than the 85th percentile, "overweight": 85th to less than the 95th percentile, and "obese": Equal to or greater than the 95th percentile [19].

### Statistics

Pearson Chi-Square Tests and Kruskal-Wallis analysis of variance (ANOVA) with multiple comparisons were used to examine differences on demographics, clinical characteristics and sleep variables, in children with and without chronic illness. Wilcoxon Signed Ranks Test was used to examine differences in the prevalence of sleep problems in the whole sample. Non-parametrical tests were chosen due to the non-normality of the data. Logistic regression analyses were used to further explore the association between chronic illness and sleep problems. In general, logistic regression analysis is considered a robust and appropriate analysis also in non-normal data. Both unadjusted (crude) analyses, as well as separate analyses adjusting for A) gender and age, B) income, education and BMI, C) parent-reported behavioural problems, and D) child-reported behavioural problems were conducted. The rationale for including behavioural problems at both waves in the regression model was to investigate the effect of both previous and co-existing behavioural problems on sleep problems. A fully adjusted analysis including all the listed potential confounders was also conducted. Finally, logistic regression analyses were conducted with the SDQ-factors as the exposure variable on subsequent sleep problems. Results are presented as odds ratios (OR) with 95 percent confidence intervals. Analyses were performed using SPSS for Windows 17, and the alpha level was set at a two-tailed 5%.

### Ethics

The study was approved by the National Data Inspectorate and the Regional Committee for Medical and Health Research Ethics in western Norway. Written informed consent was obtained from all parents included in this study. Participants received no payment to participate.

## Results

### Sample characteristics

There were significantly more boys than girls in the chronic illness group, a larger proportion was overweight/obese, and they were more likely to have a lower family income (Table 2). Children with chronic illness also reported significantly higher levels of emotional and behavioural problems at both waves compared to the no chronic illness group. No significant differences were found on age or parental education between the two groups.

**Table 2:** Demographic and clinical characteristics in children with and without chronic illness at wave 2.

Characteristics	No chronic illness	Chronic illness	*P*-value
N	3730	295	
Girls, % (n)	53.3 (1988)	42.7 (126)	< 0.001
			
Wave 1			
Age*	8.27 (8.24-8.30)	8.23 (8.13-8.33)	.47
Emotional and behavioural problems (SDQ Parents-reported)*			
Emotion	1.16 (1.11-1.21)	1.89 (1.65-2.13)	<.001
Conduct	0.82 (0.78-0.86)	1.16 (0.99-1.33)	<.001
Hyperactivity	2.40(2.33-2.46)	3.27 (2.98-3.57)	<.001
Peer	1.16 (1.11-1.21)	1.89 (1.65-2.13)	<.001
Total	5.16 (5.02-5.30)	7.77 (7.03-8.50)	<.001
			
Wave 2			
Body-mass index, % (n)			
Boys			<.001
Underweight	4.5% (82)	3.2% (6)	
Healthy weight	81.0% (1486)	73.8% (138)	
Overweight	9.5% (175)	10.7% (20)	
Obese	5.0% (91)	12.3% (23)	
Girls			.008
Underweight	6.2% (133)	4.2% (6)	
Healthy weight	80.6% (1730)	72.7% (104)	
Overweight	9.1% (196)	14.7% (21)	
Obese	4.1% (88)	8.4% (12)	
Economy, n (%)			.013
Good	68.4 (3124)	64.2 (239)	
Medium	29.0 (1326)	30.6 (114)	
Poor	2.6 (120)	5.1 (19)	
Education mother, n (%)			.31
Primary	8.3 (368)	10.7 (37)	
Secondary	39.5 (1746)	38.8 (134)	
College/University	52.1 (2303)	50.4 (174)	
Education father, n (%)			.74
Primary	8.2 (374)	8.1 (30)	
Secondary	37.7 (1712)	39.7 (147)	
College/University	54.0 (2452)	52.2 (193)	
Emotional and behavioural problems (SDQ - Child-reported)*			
Emotion	1.61 (1.56-1.66)	2.16 (1.95-2.38)	<.001
Conduct	1.05 (1.02-1.09)	1.22 (1.08-1.36)	.028
Hyperactivity	2.52 (2.46-2.57)	3.08 (2.86-3.31)	<.001
Peer	1.09 (1.05-1.14)	1.70 (1.50-1.90)	<.001
Total	6.27 (6.14-6.41)	8.17 (7.59-8.76)	<.001

* Data presented as mean (95% CI)

### Chronicity of sleep problems

**Figure 1:**
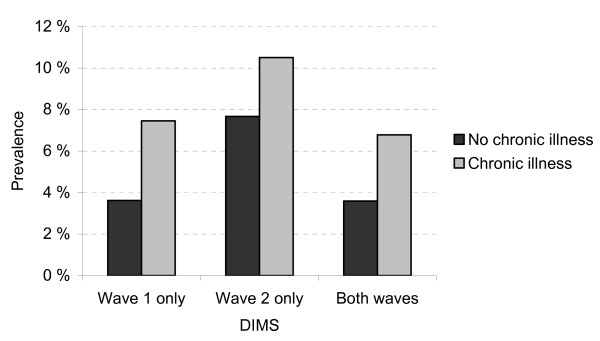
**Chronicity of sleep problems in children with and without chronic illness**. DIMS = difficulties initiating and/or maintaining sleep.

Overall, sleep problems increased significantly during the 4 year-period (8.1% to 12.3%, *Z *= 7.35, p < .001), with an increase from 15.3% to 18.8% in the chronic illness group and from 7.6 to 11.8% in the non-chronically ill group. The prevalence of chronic sleep problems (DIMS at both waves) was significantly higher among children with chronic illness (6.8%) compared to children with no chronic illness (3.6%) (χ^2 ^= 23.54, *df *= 3, *p *< .001, Figure 1).

**Figure 2:**
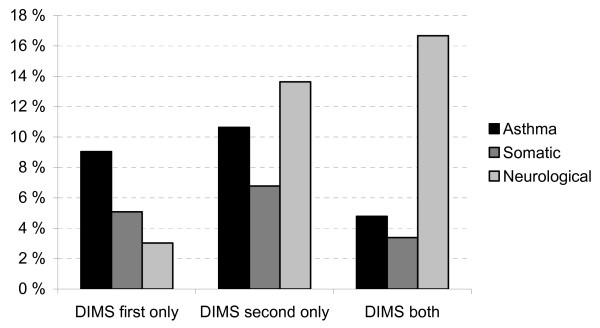
**Sleep problems at wave 1 and/or 2 in subgroups of chronic illness**. DIMS = difficulties initiating and/or maintaining sleep.

Sleep problems reported only at wave 1 was also significantly higher in the chronic illness group compared to their healthy peers (7.5% vs. 3.6%, OR = 2.52, 95%: CI 1.58-4.01), as also was the case for sleep problems only at wave 2 (10.5% vs. 7.7%, OR = 1.67, 95%: CI 1.14-2.47). Among the children with chronic illness, children with neurological disorders were more likely to have chronic sleep problems compared to children with either asthma or somatic disorders (χ^2 ^= 55.60, *df *= 6, *p *< .001, Figure 2). There were no differences in remission rates between children with and without chronic illness (38.2% vs. 46.3%, (χ^2 ^= 1.06, *df *= 2, *p *= .59).

### Predictors of sleep problems

Logistic regression analyses showed that children with a chronic illness reporting sleep problems in wave 1 had a six-fold increased risk of also having sleep problems at wave 2 (OR= 6.04, 95% CI: 2.96-12.33). Adjusting for potential confounders, including demographics, BMI, and emotional and behavioural problems, reduced the effect to OR = 5.41 (95% CI: 1.59-18.40, Table 3).

**Table 3:** Sleep problems in wave 1 as a predictor of sleep problems in wave 2, adjusting for potential confounders

	Odds-ratio	95% CI
Unadjusted (Sleep problems in Wave 1)	6.04	2.96-12.33
A Gender, age	6.35	3.05-13.20
B Income, education, and BMI (Wave 2)	6.10	2.62-14.21
C Parent -reported behavioural problems (Wave 1)	4.57	2.04-10.23
D Child-reported behavioural problems (Wave 2)	4.77	2.01-11.36
Fully adjusted model*	5.41	1.59-18.40

* Adjusting for all the confounders listed above (A+B+C+D).

To further explore the independent effect of emotional and behavioural problems, separate analyses were conducted with the SDQ as the exposure variable. As detailed in Table 4, both hyperactivity and emotional problems at wave 1 significantly predicted sleep problems in wave 2 in the unadjusted analyses. These effects remained significant when adjusting for sleep problems in wave 1 (hyperactivity problems: OR= 1.38, 95% CI: 1.13-1.69, and emotional problems: OR= 1.28, 95% CI: 1.08-1.51). Conduct problems and peer relationship problems were unrelated to subsequent sleep problems.

**Table 4:** Behavioural and emotional problems (parent-reported in wave 1) as predictors of sleep problems in wave 2.

	Unadjusted		Adjusting for sleep problems (wave 1)	
	Odds-ratio	95% CI	Odds-ratio	95% CI

Emotional problems	1.33	1.13-1.55	1.28	1.08-1.51
Conduct problems	1.16	0.90-1.51	1.04	0.79-1,37
Hyperactivity problems	1.30	1.08-1.56	1.38	1.13-1.69
Peer problems	1.02	0.84-1.22	1.03	0.85-1,24

## Discussion

The aim of the current study was to examine the chronicity and predictors of sleep problems in children with chronic illness compared to their healthy peers. Overall, the prevalence of sleep problems in both children with and without chronic illness increased from wave 1 to 2. Children with chronic illness had a higher rate of both chronic and acute sleep problems. Sleep problems at wave 1 was the strongest predictor of subsequent sleep problems. In addition, hyperactivity and emotional problems were smaller but significant risk factors.

While prospective studies of sleep problems in children in general have yielded mixed results on chronicity [9-11], the current study indicates that children have more problems initiating and maintaining sleep as they enter early adolescence, both in the chronic illness and non-chronic illness group. Being the first study to explore the course of sleep problems in children with chronic illness, the current findings show that both persistent and transient sleep problems are significantly more common in children with a chronic illness compared to healthy children. As such, the current study extends on previous cross-sectional evidence of sleep problems being more common in children with chronic illness [18].

There are several potential factors that may explain the increased persistency of sleep problems in the chronic illness group. Having a neurological disorder greatly increased the risk of developing chronic sleep problems. General risk factors, such as sociodemograhic factors and BMI, were found to be more prevalent in the chronic illness group, but only slightly reduced the risk of sleep problems at wave 2, and could hence not account for the high rate of sleep problems in the group as a whole. [20]. In a previous report from the same study [18], behavioural and emotional problems were found to account for most of the sleep problems in children with chronic illness. However, due to the cross-sectional nature of that study, no conclusions could be drawn about directions of causality. In the current study, we show that emotional and behavioural problems are independent risk factors for later sleep problems. As emotional problems was one of the strongest predictors of later sleep problems one potential mechanism of this association may be through increased worry at bedtime, which may delay sleep onset and increase night-time awakenings in the child. In sum, these findings emphasize the need for early detection of emotional and behavioural problems in this population.

There are several limitations to the present study. Chronic illness was assessed by parent report only, without medical verification of the diagnosis. Difficulties initiating or maintaining sleep were assessed by a joint variable, making it difficult to examine each construct separately and to assess the importance of the finings, and we also had no measure of the severity and duration of the sleep problems. Although not a validated measurement of sleep problems, we still consider that its inclusion in the present study design adds valuable information in a field and age cohort in which the focus on sleep problems has been virtually non-existing in epidemiological research. Unfortunately, the operationalization of insomnia and sleep problems has been extremely diverse in the general sleep literature, causing problems when comparing results across studies [21,22]. Therefore, future studies should seek to employ validated instruments based on agree-upon diagnostic criteria when assessing sleep problems to facilitate study comparisons. Also, we had no measure of symptoms of obstructive sleep apnoea (OSA), which previously has been linked to obesity in children in general. OSA may be one potential mechanism through which obesity may contribute to increased sleep problems in this group. Another limitation is number of dropouts from wave 1 to 2, and we unfortunately have no information as to why these families did not participate in the longitudinal study. Also, several of the potential factors that could affect the relationship between sleep and chronic illness were only assessed in the second wave, and hence could not be used as predictors of chronicity of sleep problems. Finally, children with mental retardation were included in chronic illness group, the reason being the high degree of overlap between parent-reported mental retardation and having another neurological disorder. As such, excluding mental retardation from the CI-group would both have considerably reduced the sample size as well placed a substantial amount of children with CI in the healthy comparison group.

Increased awareness of the course of sleep problems over time is important for both clinicians, as well as to caregivers. In contrast to the common belief that children often will outgrow their sleep problems, the current study shows that this may not be the case, especially in children with chronic illness, thereby emphasizing the need to develop treatment strategies for this group of children. In addition, previous studies in the adult population have shown that even small improvements in sleep quality may yield noticeable relief in other co-existing symptoms (such as pain or fatigue) [23]. There is now substantial evidence that behavioural interventions are efficacious in treating sleep problems in children [24], with more than 80% showing clinically significant and lasting improvements. In addition, pharmacological interventions may be beneficial in subgroups of children with CI. In cases where mental retardation and hyperactivity co-exist with other chronic illnesses, circadian rhythm sleep disorders plays an important role in the aetiology of the sleep problems, in which adequately timed melatonin has shown to effectively relieve chronic sleep problems [25-28]. When also considering that improved sleep may have positive effects on both psychological, academic and possibly physiological variables [29], we consider it especially important that sleep problems in children with chronic illness are detected and managed adequately. Because disrupted sleep in children also influences other members of the family and remains a primary concern for many parents and caregivers [30], the quality of life for the child as well as her or his family as a whole may improve following treatment of sleep problems.

## Competing interests

The authors declare that they have no competing interests.

## Authors' contributions

BS and MH carried out the statistical analyses and drafted the manuscript. AJL and KMS participated in the design of the study, and AJL provided critical comments in drafting the manuscript. IE provided categorization of chronic illness conditions, and aided in the drafting process. All authors read and approved the final manuscript.
